# Editorial: Non-pharmacological approaches for cardiovascular health in underrepresented populations

**DOI:** 10.3389/fcvm.2025.1718238

**Published:** 2025-11-04

**Authors:** Ladislav Batalik, Jing Jing Su, Demosthenes Panagiotakos, Stefanos Tyrovolas, Yao Jie Xie

**Affiliations:** ^1^ Department of Rehabilitation, University Hospital Brno, Brno, Czechia; ^2^ Department of Physiotherapy and Rehabilitation, Faculty of Medicine, Masaryk University, Brno, Czechia; ^3^ Rehabilitation Clinic, Faculty of Medicine, Masaryk University, Brno, Czechia; ^4^ Department of Public Health, Faculty of Medicine, Masaryk University, Brno, Czechia; ^5^ School of Nursing, Tung Wah College, Hong Kong, Hong Kong SAR, China; ^6^ School of Health Sciences, Harokopio University, Athens, Greece; ^7^ Department of Nutrition and Food Studies, George Mason University, Fairfax, VA, United States; ^8^ School of Nursing, Faculty of Health and Social Sciences, The Hong Kong Polytechnic University, Hong Kong, Hong Kong SAR, China; ^9^ Research, Innovation and Teaching Unit, Parc Sanitari Sant Joan de Déu, Sant Boi de Llobregat, Spain; ^10^ Instituto de Salud Carlos III, Centro de Investigación Biomédica en Red de Salud Mental, Centro de Investigación Biomédica en Red de Salud Mental (CIBERSAM), Madrid, Spain; ^11^ Research Centre for Chinese Medicine Innovation, The Hong Kong Polytechnic University, Hong Kong, Hong Kong SAR, China

**Keywords:** cardiovascular disease prevention, non-pharmacological interventions, health equity, cardiac rehabilitation, underrepresented populations, lifestyle modification

**Editorial on the Research Topic**
Non-pharmacological approaches for cardiovascular health in underrepresented populations

Cardiovascular disease (CVD) remains a leading driver of preventable illness and death, with a disproportionate burden carried by underrepresented populations—racial and ethnic minorities, people with lower socioeconomic status, older adults, women (including post-menopause), and individuals living with multimorbidity. Pharmacotherapy is necessary but not sufficient to close these gaps. Non-pharmacological strategies— covering lifestyle, education, behavior change, community design, service delivery, and technology—offer holistic, low-risk, and sustainable paths to prevent disease, improve outcomes, and advance equity.

This Research Topic gathers studies that move the conversation beyond what works to what works, for whom, in which contexts, and how to scale it fairly. Together, the contributions highlight three connected levers for change: (1) acting on risk patterns rather than isolated factors; (2) tailoring interventions to contexts, identities, and life stages; and (3) bridging the ongoing policy-to-practice gap, particularly in limited-resource settings.

## Action patterns and places, not just single risks

Two contributions from the ATTICA cohort underscore how risks cluster within social and environmental contexts. Damigou et al. estimate the preventable burden of CVD when lifestyle, psychosocial, clinical, and socioeconomic factors are addressed together, highlighting the substantial benefits possible when we target patterns of exposure rather than single behaviors (Damigou et al.). In addition, Sigala et al. probe the built environment, showing that long-term urban–rural differences in CVD incidence are partly explained by modifiable lifestyle behaviors. The message is clear: where people live—and the opportunities/constraints their neighborhoods place on them—shapes cardiovascular trajectories. Effective prevention must therefore combine individual counseling with broader levers such as healthy city design, green and active transport, and equitable access to nutritious food and safe physical-activity spaces (Sigala et al.).

## Tailor and co-create interventions with the people they serve

Several articles demonstrate the value of designs that meet people where they are—biologically, culturally, and socially. Wu et al. synthesize randomized evidence on exercise modalities for individuals at increased CVD risk, comparing aerobic, interval, resistance, and combined training (Wu et al.). Their network meta-analysis not only ranks options for improving arterial stiffness and blood pressure but also points to the importance of intensity and combination—practical insights for clinicians personalizing exercise prescriptions within rehabilitation and primary prevention.

Chen et al. explore a novel contextual question—whether high-intensity interval training in a cold environment influences arterial stiffness and cerebral hemodynamics among sedentary young women after COVID-19. Although focused on a specific subgroup, the study speaks to a broader principle: physiologic state, recovery status, and environmental conditions work together, and protocols may need adaptation for groups typically underrepresented in exercise trials (Chen et al.).

Gender-responsive education is another main theme. Carson et al. evaluate “Cardiac College for Women”, finding high acceptability and ease of implementation across sites. Women frequently encounter barriers to standard cardiac rehabilitation (CR)— family care responsibilities, cultural norms, and the historical male-centric design of CR programs. Specially designed curricula and delivery models can enhance engagement, health literacy, and self-management, offering a replicable path for other identity-tailored CR services (e.g., by age, language, or cultural community).

Finally, even when pharmacotherapy is indicated, behavior and systems determine whether benefits are realized. Villalobos-Pedroza et al. report real-world adherence and risk-factor control after ST-elevation myocardial infarction in Mexico, revealing substantial room for improvement. Their findings remind us that “non-pharmacological” is not opposed to medication; rather, it includes the behavioral, educational, and service-design elements that enable people to start and maintain evidence-based care.

## Mitigate the knowing–doing gap in low- and middle-income settings

Mendis and Graham bring attention to the continuing challenge that global NCD targets remain unmet, particularly in low- and middle-income countries. They lay out a pragmatic way forward: prioritize affordable, high-impact interventions within primary health care; build national capacity for implementation; and use the WHO 2023–2030 roadmap as a framework. Their synthesis is a timely reminder that evidence alone does not reduce inequities; delivery systems do.

## Implications

Taken together, these studies offer a clear plan for equitable cardiovascular health by shifting from single risks to risk clusters, using population-level metrics and mediation models to prioritize multicomponent strategies across age, sex, and socioeconomic strata; designing with—not for—communities through culturally meaningful education, built-in social support, and removal of practical barriers such as childcare, transportation, and work schedules; personalizing movement by tailoring exercise prescriptions to modality, intensity, comorbidity, recovery state, and environmental context, and integrating these protocols into CR and primary care. Efforts should also focus on making places healthier by aligning public health with urban planning to foster active living and reduce cardiometabolic risks at the neighborhood level.

In low- and middle-income countries, there is a need to strengthen implementation by expanding low-cost interventions through primary care, building up staff and data systems, and monitoring fidelity, reach, and equity outcomes. Technology should be used judiciously and wherever appropriate—spanning low-end eHealth, telerehabilitation, and remote monitoring to high-end AI-based risk stratification—to reduce long-term healthcare costs for individuals, health systems, and society. Finally, future research must measure what matters for fair treatment by reporting subgroup effects and equity-focused outcomes such as uptake, adherence, continuity, and financial burden, alongside traditional clinical endpoints.

## A call to action

Non-pharmacological approaches are not secondary to CVD care; they are central to health equity. The contributions in this collection together show argue for a shift: from isolated advice to integrated programs; from clinic-centric models to community-based ecosystems; from efficacy trials to implementation strategies that work in the real-world complexity. Success will require multi-disciplinary partnerships—clinicians, rehabilitation specialists, public-health practitioners, planners, data scientists, and, crucially, the communities most impacted.

Looking ahead, judicious use of technology will amplify impact: low-end eHealth, telerehabilitation, and remote monitoring will extend reach to underserved populations; decision support and AI-enabled risk stratification will enable timely, personalized care; and interoperable platforms will strengthen team-based coordination and adherence. These tools are poised to lower long-term costs by reducing preventable events and improving continuity of care, while generating real-time data to guide adaptive, context-specific implementation. To ensure benefits are widely shared, solutions should be inclusively designed (language, culture, digital literacy), rigorously evaluated for safety and fairness, and integrated within trusted community settings.

We hope this Research Topic encourages further scholarship that is methodologically rigorous, mindful of context, and equity-first. The way forward is to combine strong science with intentional design and delivery—so that the promise of non-pharmacological prevention and rehabilitation is realized by those who have long been left behind.

